# Association between Dietary Potassium Intake and Nonalcoholic Fatty Liver Disease and Advanced Hepatic Fibrosis in U.S. Adults

**DOI:** 10.1155/2024/5588104

**Published:** 2024-07-15

**Authors:** Hao-Kai Chen, Qi-Wen Lan, Yu-Jia Li, Qing Xin, Run-Qi Luo, Jun-Jie Wang

**Affiliations:** ^1^ Department of Infectious Diseases The Second Affiliated Hospital Guangzhou Medical University, Guangzhou, China; ^2^ The Third School of Clinical Medicine Guangzhou Medical University, Guangzhou, China; ^3^ The Second School of Clinical Medicine Guangzhou Medical University, Guangzhou, China

## Abstract

**Introduction:**

The correlation between potassium and nonalcoholic fatty liver disease (NAFLD) is currently still poorly understood. We conducted this study to explore the correlation between dietary potassium intake and NAFLD, as well as advanced hepatic fibrosis (AHF). The study also sought to identify any potential interactions.

**Methods:**

The data employed in this study were obtained from the National Health and Nutrition Examination Survey (NHANES) program, encompassing a period from 2007 to 2018. Employing the multiple logistic regression analysis, we evaluated the association of dietary potassium intake with NAFLD and AHF. Subsequently, stratification analysis, based on demographic variables, was constructed so as to assess the stability of the results. In addition, potential interaction effects were assessed by interaction tests.

**Results:**

A total of 9443 participants were included in the analysis. The mean age of the participants was 50.4 years, and their daily mean dietary potassium and vitamin C intake was 2556.49 mg and 82.93 mg, respectively. Following comprehensive statistical analyses, the findings indicated a negative correlation between dietary potassium intake and both NAFLD and AHF. Participants in Q4 group with dietary potassium intake exhibited a 31% and 42% reduction in the odds of developing NAFLD and AHF, respectively, in comparison to Q1 group. An interaction effect of dietary vitamin C intake was observed in the association between dietary potassium intake and NAFLD. The results imply that high dietary vitamin C intake augment the inverse relationship between dietary potassium intake and NAFLD.

**Conclusion:**

Dietary potassium intake was found to have an inverse association with the odds of both NAFLD and AHF. The association between dietary potassium intake and NAFLD was amplified by the presence of vitamin C in the diet.

## 1. Introduction

Nonalcoholic fatty liver disease (NAFLD), a prevalent metabolic liver disease, is characterized by the accumulation of fat in the liver, excluding the influence of other liver disease factors and significant alcohol consumption [1]. Patients with NAFLD have hepatic steatosis in at least 5% of hepatocytes on liver biopsy [2]. Fibrotic progression leads to advanced hepatic fibrosis (AHF) in NAFLD [3]. The incidence of NAFLD has increased rapidly in the last few years [4]. Moreover, it is often considered a highly relevant potential risk factor for metabolic illnesses, such as type 2 diabetes and dyslipidemia [1]. Recent studies indicated a gradual increase in the prevalence of NAFLD among adults and children as the incidence of metabolic diseases such as obesity and type 2 diabetes increases, which could result in a global prevalence of NAFLD reaching 35% within the forthcoming decade [5].

Potassium ions are an essential element in daily dietary intake, primarily from fruits and vegetables [6]. Low dietary potassium intake has been reported to be correlated with various metabolic disorders [7]. Furthermore, the danger of CVD has been cited to rise with a reduction in dietary potassium intake [8]. However, as a common metabolic liver disease, NAFLD is related to dietary potassium intake. In a cross-sectional community study of mid-aged and elderly adults in China, low serum potassium levels were demonstrated clearly to be significantly correlated with the frequency of NAFLD in these two populations [7]. In children with NAFLD, there is a negative correlation between serum potassium levels and advanced fibrosis of the disease [9]. What's more, there are findings that indicate that people with NAFLD typically consume less potassium than healthy individuals [10]. In a study conducted by Leila Azadbakht, a high dietary potassium intake was observed to increase body's antioxidant capacity and reduce the risk of oxidative stress, which had the same effect as vitamin C [11, 12]. The similar role played in reducing oxidative stress suggests that dietary potassium and vitamin C may be potentially related.

Vitamin C, scientifically referred to as ascorbic acid, is predominantly sourced from fruits and vegetables [13]. As a necessary antioxidant, vitamin C has the ability to neutralize free radicals and reactive oxygen species [12]. Apart from this, it is also involved in regulating hepatic and circulating lipid homeostasis in humans [14]. Currently, several studies have shown the preventive effect of vitamin C on NAFLD. In a cross-sectional study involving a substantial cohort, the level of vitamin C people consumed in daily food had been proved to be negatively affected by the severity of NAFLD [15]. Furthermore, vitamin C may be more effective in preventing NAFLD than treating the disease, according to a recent animal study [16].

Although several past studies have reported a possible association between dietary potassium intake and NAFLD, such as serum potassium level was significantly associated with the prevalence of NAFLD in middle-aged and older Chinese adults and with advanced fibrosis in children with NAFLD [7, 9], it is still important to investigate the association in the U.S. population. In addition, there is limited research on the association of dietary potassium intake with AHF. Furthermore, we attempted to explore the potential interactive role that dietary vitamin C plays in the association between dietary potassium intake and both NAFLD and AHF, which has not been found in past studies yet. Therefore, we conducted this study with the aim of exploring the association between dietary potassium intake and both NAFLD and AHF and the potential interactions.

## 2. Materials and Methods

The methods of this study follow the first author's previously published study and the methods description partly reproduces their wording [17, 18].

### 2.1. Data Sources and Study Design

The data employed in this study were derived from the National Health and Nutrition Examination Survey (NHANES) program, encompassing a period from 2007 to 2018. NHANES is a national study that investigates the nutrition and health of Americans. Data pertaining to participant demographics and health status was meticulously collected by professionals through the use of questionnaires and health interviews. To further enhance the comprehensiveness of the data, laboratory tests were also conducted. The participants' nutritional condition was ascertained via a 24-hour dietary recollection. Health assessments and the gathering of blood specimens were executed in a transportable examination facility (MEC). Data from participants that were labeled as missing, declined, or unknown on the NHANES website were regarded as incomplete information.

**Figure 1:**
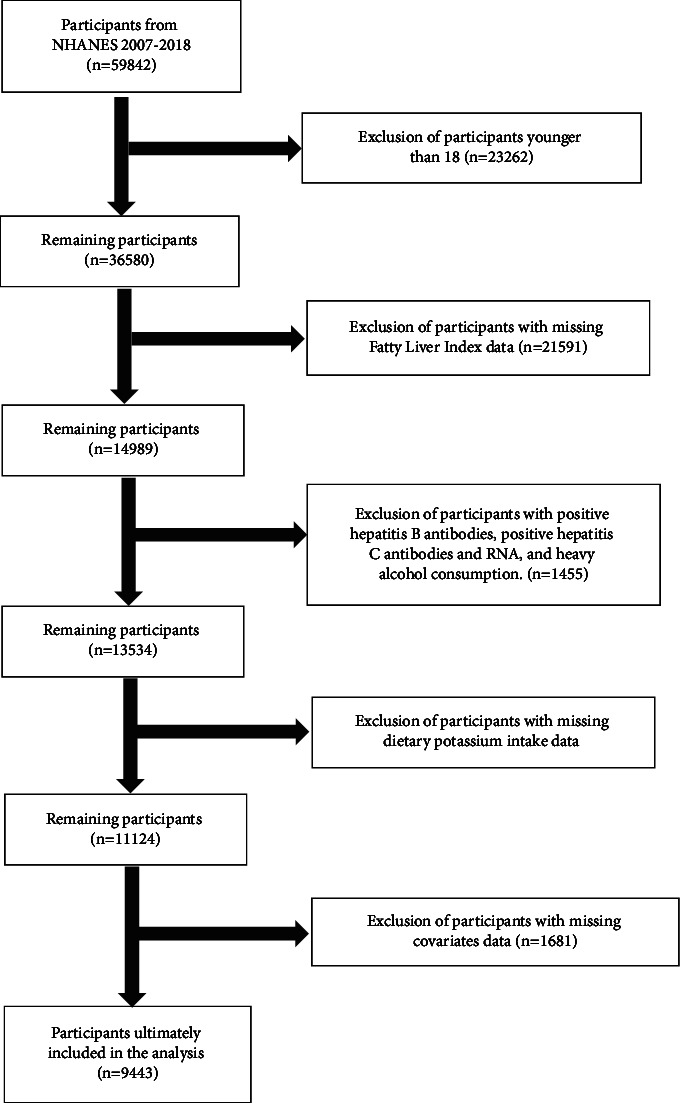
Flowchart for inclusion and exclusion of participants.

To mitigate the potential for skewed results due to exceptional participant conditions, the study excluded individuals who were under the age of 18, tested positive for Hepatitis B antibodies, tested positive for Hepatitis C antibodies or RNA, or exhibited high alcohol consumption (surpassing 30 g/d for males and 20 g/d for females). Furthermore, we excluded participants who had incomplete data on the Fatty Liver Index (FLI), Nonalcoholic Fatty Liver Disease Fibrosis Score (NFS), dietary potassium consumption, and covariates. Ultimately, a total of 9443 participants were incorporated in the analysis. The procedure for screening participants is depicted in Figure 1.

### 2.2. Definition of Exposure

The FLI is a widely recognized indicator employed to predict NAFLD [19, 20]. The research utilized the FLI as a criterion to diagnose NAFLD. Participants who had an FLI score of 60 or above were classified as having NAFLD [21]. The NFS, a noninvasive method, was employed to identify advanced hepatic fibrosis [22]. In the study, participants having NAFLD with an NFS greater than 0.676 were considered to suffer from AHF. The calculations for FLI and NFS have been detailed in previous studies [21, 22].

### 2.3. Covariates

In order to build multiple logistic regression models and eliminate the influence of possible elements on the results, variables such as age, sex, ethnicity, level of education, family economic status, tobacco usage, employment activities condition, leisure activities condition, dietary energy, protein, alcohol consumption, high blood pressure condition, diabetes condition, and biochemical markers, encompassing uric acid, fasting sugar levels, total cholesterol, glycosylated hemoglobin, and HDL cholesterol, were selected as covariates for examination. The ethnicity of the participants was classified into five distinct groups, which include Mexican American, other Hispanic, among others. Educational attainment is categorized into three distinct levels: below high school, high school, and above high school. The family income-to-poverty ratio was divided into three categories: less than 1, between 1 and 3, and more than 3. The smoking status of individuals was categorized as never smoked, former smoker, and now smoking. Participants' activities, both professional and recreational, were evaluated using four levels: “No Activity,” “Vigorous,” “Moderate,” and “Both Vigorous and Moderate.” Individuals on medication for high blood pressure or with a previous/current diagnosis of hypertension were identified as hypertensive.

### 2.4. Statistics Analysis

In characterizing participants, categorical variables were described by using weighted percentages (%). We utilized “mean ± standard deviation (SD)” or “median (interquartile range)” for the characterization of continuous variables. For categorical variables, the chi-square (*χ*^2^) test was employed to evaluate their statistical significance, while for continuous variables, the Kruskal‒Wallis test was utilized. The correlation between dietary potassium intake and NAFLD and AHF was investigated through multiple logistic regression analysis, and adjusted models were established grounded on covariates. In multiple logistic regression, dietary potassium intake was equally divided into four groups. In order to examine the correlation with dietary potassium intake considered as a continuous variable, *Z*-score was calculated, and odds ratios (OR) for NAFLD and AHF associated with each SD increment in dietary potassium consumption were reported. Following that, we illustrated the correlation by sketching a fitting curve, derived from the adjusted model 5 (data were transformed using natural logarithm).

Moreover, stratified examinations were established based on age, sex, ethnicity, education attainment, and the family income-to-poverty ratio to scrutinize the steadiness of the association between dietary potassium intake (per SD increase) and NAFLD or AHF. The stratified analyses were adjusted based on model 5. Interaction tests were conducted on covariates to explore the interaction factors associated with the results. Two adjusted models were also established in the interaction test to eliminate the influence of confounding elements. The 95% confidence intervals were computed in this investigation. For all analyses conducted, a *P* value of less than 0.05 was deemed to indicate statistical significance. The statistical package R was used to extract the data, and Empower Stats software was employed to conduct statistical analysis of the data.

## 3. Results

### 3.1. Baseline Characteristics of Participants

Data from NHANES between 2007 and 2018 were instrumented in this research, and 9443 participants were comprised in the analysis. NAFLD was diagnosed in 4221 participants. The baseline characteristics of the participants are exhibited in Table 1. Participants with NAFLD tended to be older, male, nonHispanic White, lower education level, poorer household economic status, current or former smokers, and they were more likely to engage in intense work activities, be lacking in recreational activities, and have a higher BMI or waist circumference. In addition, participants with NAFLD had higher rates of hypertension and diabetes. More importantly, participants with NAFLD were observed to have lower levels of dietary potassium intake (2527.94 ± 1035.22 vs 2579.56 ± 1089.59, *P* = 0.019). Overall, statistically significant differences were observed in all participant characteristics.

### 3.2. Association between Dietary Potassium Intake and NAFLD and AHF

**Figure 2:**
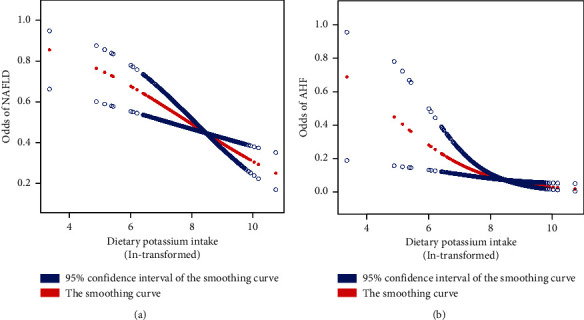
Smoothing curve fitting plot. NAFLD, nonalcoholic fatty liver disease; AHF, advanced hepatic fibrosis. (a) The negative association between dietary potassium intake and NAFLD. (b) The negative association between dietary potassium intake and AHF.

The association of dietary potassium intake with NAFLD and AHF is shown in Table 2. Dietary potassium intake was divided equally into 4 groups (Q1: 14.5–1831, Q2: 1831.5–2401, Q3: 2401.5–3106.5, Q4: 3107–22665.5). A crude model and 5 adjusted models were included in the multiple logistic regression analysis. Across all models, we observed a negative and statistically significant correlation between dietary potassium intake and both NAFLD and AHF. In the fully adjusted model (adjusted model 5), compared with Q1 group (lowest), participants in Q4 group (highest) were 31% (OR = 0.69, 95% CI 0.54, 0.88, *P* < 0.05) less likely to have NAFLD and 42% (OR = 0.58, 95% CI 0.38, 0.88, *P* < 0.05) less likely to have AHF in the dietary potassium intake. Furthermore, with each SD increase in dietary potassium intake, participants' risk of NAFLD and AHF decreased by 12% (OR = 0.88, 95% CI 0.80, 0.96, *P* < 0.05) and 19% (OR = 0.81, 95% CI 0.69, 0.96, *P* < 0.05), respectively. The smoothed curve fit diagram, demonstrating a negative correlation between dietary potassium intake and both NAFLD and AHF, is presented in Figure 2.

### 3.3. Stratified Analysis

**Figure 3:**
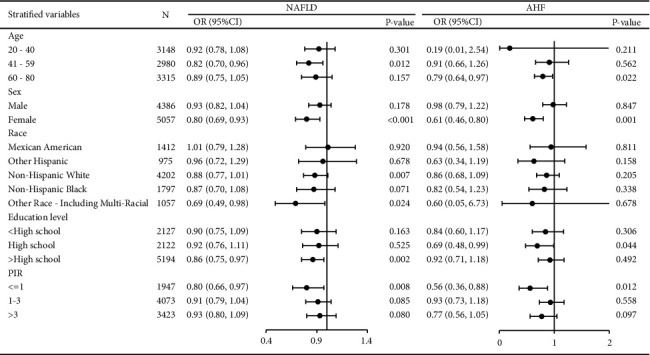
Stratified analysis of the association between dietary potassium intake and NAFLD and AHF. NAFLD, nonalcoholic fatty liver disease; AHF, advanced hepatic fibrosis.

The stratified variables in the stratified analysis encompass age, sex, ethnic, level of education level, and the family income-to-poverty ratio. As depicted in Figure 3, the negative correlation between dietary potassium intake and both NAFLD and AHF is consistently observed across almost all participant groups.

### 3.4. Interaction Analysis

The interaction of dietary vitamin C intake and dietary potassium intake on NAFLD is shown in Table 3. In the low dietary vitamin C intake group, it was difficult to observe a negative correlation between dietary potassium intake and NAFLD. However, potassium intake showed a significant negative association with NAFLD in the high dietary vitamin C intake group. Interaction effects were observed to be statistically significant in all 3 models.

Table 4 shows the interaction of dietary vitamin C and potassium intake on AHF. A negative correlation was observed between potassium intake and AHF in both groups with high and low dietary intakes of vitamin C. However, no statistically significant interaction effect could be observed in any of the three models.

## 4. Discussion

Our research, based on the analysis of NHANES data from 2007 to 2018, indicates dietary potassium intake is negatively associated with both NAFLD and AHF. Moreover, after stratifying the analyses by characteristic demographic variables, we found that the association showed significant stability. Additionally, in all models, dietary vitamin C intake interacted with dietary potassium intake in reducing the rate of NAFLD, suggesting that their joint effect on NAFLD was superior to the aggregate of the two alone. However, their interaction was not observed in AHF.

Several past studies have also reported the association between potassium and NAFLD. Kan Sun and others revealed that low serum potassium was linked with the prevalence of NAFLD in middle-aged and elderly adults in China [7]. Adam Tabbaa found that serum potassium was negatively correlated with the severity of fibrous degeneration in NAFLD among children in Italian population [9]. Our findings were consistent with the above study. It is interesting to note that a study from Korea showed that there were certain associations between dietary potassium intake and the incidence of ultrasound-diagnosed NAFLD, but they failed to observe a statistical correlation in the end [23]. Adjustment for dietary energy intake in multivariable logistic regression may be a key factor contributing to the different results of the above studies. The study from Korea claimed that the association between dietary potassium and NAFLD disappeared after adjustment for dietary energy intake, while the studies from China and Italy did not adjust. In addition, the age, ethnicity, and dietary differences of the participants may have been factors that accounted for the differing conclusions.

There are several possible mechanisms of the development of NAFLD and its advanced fibrosis due to low dietary potassium intake. Above all, low dietary potassium intake increases the expression and activity of angiotensin-converting enzymes and enhances the systemic responsiveness to angiotensin II, that is, the development of RAS dysregulation [24, 25]. RAS dysregulation plays a dominant role in tissue damage and advanced fibrosis in chronic liver disease [26]. Overexpression of ANG II induces the activation of HSCs in vivo, which contributes to the genesis of hepatic steatosis and advanced fibrosis [27, 28]. In addition, in Mark C. Houston's study, it was concluded that increasing dietary potassium intake led to an improvement in insulin sensitivity and a reduction in oxidative stress and inflammation [29]. This is because potassium ions can induce cell depolarization and thus insulin secretion by pancreatic beta cells [30]. As a causative factor in NAFLD, oxidative stress may cause cellular dysfunction, which is the origin of liver's adverse response to fat accumulation, resulting in liver metabolic damage as well as NASH progression [31, 32]. Additionally, inflammation in adipose tissue is known to drive the process of NASH [33].

Epidemiological and clinical findings indicate that there is a high proportion of patients with NAFLD who currently exhibit serum vitamin C deficiency [34]. As the sole antioxidant capable of resisting lipid oxidation, high-dose vitamin C treatment not only prevents the build-up of triglycerides within the liver but also protects it from infiltration by inflammatory cells and inflammatory cytokines [35, 36]. A study by Luo et al. demonstrated that increasing the consumption of vitamin C may replenish albumin deficiency in NAFLD patients and may lead to an improvement in liver function [14]. Moreover, Alexis Laurent believes that increased lipid peroxidation and ROS production are the key risk factors in the progression of NAFLD [37]. Supplemental vitamin C lowers the circulation of lipid concentrations and reduces the production of ROS in mitochondria, which delays the development of NAFLD and AHF [38, 39].

The interaction that dietary potassium and vitamin C intake play in reducing the risk of developing NAFLD may be explained by inflammatory and oxidative stress pathways. It has been shown that both dietary vitamin C and potassium may reduce oxidative stress and inflammation, and the joint effect may have a mutually amplifying effect [30, 39]. In addition, past studies have shown an association between systemic inflammation and hepatic steatosis [40, 41]. Nevertheless, more in-depth mechanistic studies are still lacking, and further work is needed on the interaction between dietary vitamin C and potassium. A study from the U.S. Department of Agriculture suggests that plant-based foods such as fruits/vegetables and grain-based dishes, and animal-based foods including dairy and meat/poultry are the main sources of dietary potassium intake for U.S. adults [42]. Fruits and vegetables are the richest source of dietary vitamin C. According to the results of our study, increasing the intake of the above foods may have an important role in the prevention of NAFLD and AHF.

Our research included a large, well-designed sample based on a noninstitutionalized population, which is highly representative and reliable. Indeed, while our study provides valuable insights, it is important to acknowledge its limitations. Firstly, one of the limitations of our study is that, due to its cross-sectional nature, we could not establish a causal correlation between dietary potassium intake and both NAFLD and AHF. Further longitudinal studies are needed to explore this potential causal correlation. Furthermore, since all participants were from America, the applicability of the results obtained in this study to other nationalities needs to be further investigated. Third, there is a risk that the results may be confounded by residual confounding, despite multiple adjusted models being constructed to exclude the effects of confounding factors. Due to missing and incomplete data, this study did not analyze the effect of factors such as special diets, cancer, or autoimmune diseases on the association. These limitations should be taken into account when interpreting the results of this study. Fourth, a two-day, 24-hour dietary interview was utilized in this study to collect dietary data from participants. Although the NHANES program is sufficiently rigorous, measurement errors due to recall bias may still exist. Finally, although an interaction between dietary vitamin C intake and dietary potassium intake on NAFLD has been observed, there is a lack of relevant mechanistic investigations. Therefore, further clinical studies are needed.

## 5. Conclusions

Overall, our study demonstrated a negative correlation between dietary potassium intake and both NAFLD and AHF. The intake of dietary vitamin C was found to reinforce the association between dietary potassium intake and NAFLD. Enhancing the intake of dietary potassium and vitamin C could potentially play a significant role in the prevention and management of both NAFLD and AHF.

## Figures and Tables

**Table 1:** Baseline characteristics of participants.

Characteristic	NAFLD		*P* value
	No	Yes	
*N*	5222	4221	
Demographics			
Age (year), mean ± SD	48.88 ± 18.41	52.28 ± 16.24	<0.001
Sex			<0.001
Male	2250 (43.09%)	2136 (50.60%)	
Female	2972 (56.91%)	2085 (49.40%)	
Race			<0.001
Mexican American	662 (12.68%)	750 (17.77%)	
Other Hispanic	538 (10.30%)	437 (10.35%)	
Non-Hispanic White	2261 (43.30%)	1941 (45.98%)	
Non-Hispanic Black	979 (18.75%)	818 (19.38%)	
Other race-including multiracial	782 (14.98%)	275 (6.52%)	
Education level			<0.001
<High school	1048 (20.07%)	1079 (25.56%)	
High school	1109 (21.24%)	1013 (24.00%)	
>High school	3065 (58.69%)	2129 (50.44%)	
Ratio of family income to poverty			<0.001
≤1	1020 (19.53%)	927 (21.96%)	
1–3	2146 (41.10%)	1927 (45.65%)	
>3	2056 (39.37%)	1367 (32.39%)	
Behavioral characteristics			
Smoking status			<0.001
Never	3239 (62.03%)	2257 (53.47%)	
Now	879 (16.83%)	734 (17.39%)	
Former	1104 (21.14%)	1230 (29.14%)	
Work activities status			0.022
No	3085 (59.08%)	2362 (55.96%)	
Vigorous	186 (3.56%)	164 (3.89%)	
Moderate	1180 (22.60%)	1011 (23.95%)	
Both	771 (14.76%)	684 (16.20%)	
Recreational activities status			<0.001
No	2359 (45.17%)	2499 (59.20%)	
Vigorous	475 (9.10%)	217 (5.14%)	
Moderate	1483 (28.40%)	1091 (25.85%)	
Both	905 (17.33%)	414 (9.81%)	
Dietary characteristics			
Dietary energy intake (kcal), mean ± SD	1951.52 ± 759.67	1986.73 ± 799.73	0.029
Dietary protein intake (g), mean ± SD	77.61 ± 33.48	79.85 ± 34.78	0.001
Dietary sugars intake (g), median (Q1-Q3)	95.64 (64.69–134.55)	94.15 (62.22–136.40)	
Dietary fat intake (g), mean ± SD	74.47 ± 35.86	78.51 ± 37.89	
Dietary cholesterol intake (mg), median (Q1-Q3)	227.50 (145.00–350.00)	257.50 (162.50–386.00)	
Dietary zinc intake (mg), mean ± SD	10.65 ± 5.33	10.96 ± 5.99	
Dietary sodium intake (mg), mean ± SD	3272.34 ± 1425.76	3395.77 ± 1501.12	
Dietary alcohol intake (g), mean ± SD	2.64 ± 5.95	2.25 ± 5.78	0.001
Dietary potassium intake (mg), mean ± SD	2579.56 ± 1089.59	2527.94 ± 1035.22	0.019
Dietary vitamin C (mg), median (Q1-Q3)	67.35 (33.10–118.20)	57.50 (27.25–106.65)	<0.001
Related disease conditions			
Hypertension status			<0.001
No	3489 (66.81%)	1860 (44.07%)	
Yes	1733 (33.19%)	2361 (55.93%)	
Diabetes status			<0.001
No	3813 (73.02%)	1874 (44.40%)	
Yes	639 (12.24%)	1440 (34.12%)	
IFG	392 (7.51%)	538 (12.75%)	
IGT	378 (7.24%)	369 (8.74%)	
Anthropometric measurements			
BMI (kg/m^2^), mean ± SD	25.09 ± 3.57	34.73 ± 6.27	<0.001
Biochemical indicators			
Uric acid (mg/dl), mean ± SD	5.05 ± 1.27	5.98 ± 1.42	<0.001
Fasting glucose (mg/dl), mean ± SD	102.24 ± 25.53	119.85 ± 43.50	<0.001
Glycohemoglobin (%), mean ± SD	5.57 ± 0.82	6.09 ± 1.32	<0.001
Creatinine (mg/dL), median (Q1-Q3)	0.82 (0.69–0.98)	0.85 (0.72–1.02)	<0.001
Total cholesterol (mg/dL), mean ± SD	187.71 ± 39.83	195.54 ± 42.19	<0.001
HDL cholesterol (mg/dL), mean ± SD	58.05 ± 15.45	46.82 ± 12.13	<0.001

NAFLD, nonalcoholic fatty liver disease; IFG, impaired fasting glycemia; IGT, impaired glucose tolerance; BMI, body mass index.

**Table 2:** Association between dietary potassium intake and NAFLD and AHF.

Models	NAFLD		AHF	
	OR (95% CI)	*P* value	OR (95% CI)	*P* value
Crude model				
Dietary potassium intake quartile				
Q1	Ref		Ref	
Q2	0.90 (0.80, 1.01)	0.074	0.85 (0.69, 1.04)	0.117
Q3	0.95 (0.85, 1.07)	0.423	0.80 (0.65, 0.98)	0.033
Q4	0.87 (0.78, 0.98)	0.023	0.62 (0.50, 0.78)	<0.001
Per SD increment	0.95 (0.91, 0.99)	0.019	0.83 (0.77, 0.91)	<0.001
Adjusted model 1				
Dietary potassium intake quartile				
Q1	Ref		Ref	
Q2	0.89 (0.79, 1.00)	0.060	0.87 (0.70, 1.09)	0.219
Q3	0.92 (0.82, 1.04)	0.184	0.82 (0.66, 1.03)	0.094
Q4	0.81 (0.72, 0.92)	0.001	0.69 (0.54, 0.88)	0.003
Per SD increment	0.92 (0.88, 0.96)	<0.001	0.87 (0.79, 0.96)	0.004
Adjusted model 2				
Dietary potassium intake quartile				
Q1	Ref		Ref	
Q2	0.91 (0.81, 1.02)	0.114	0.87 (0.70, 1.09)	0.218
Q3	0.94 (0.83, 1.06)	0.324	0.83 (0.66, 1.05)	0.118
Q4	0.85 (0.75, 0.96)	0.011	0.72 (0.56, 0.92)	0.009
Per SD increment	0.94 (0.90, 0.98)	0.006	0.88 (0.80, 0.97)	0.014
Adjusted model 3				
Dietary potassium intake quartile				
Q1	Ref		Ref	
Q2	0.81 (0.71, 0.93)	0.003	0.81 (0.63, 1.05)	0.109
Q3	0.77 (0.66, 0.90)	0.001	0.71 (0.53, 0.96)	0.027
Q4	0.64 (0.53, 0.77)	<0.001	0.55 (0.38, 0.80)	0.002
Per SD increment	0.83 (0.78, 0.89)	<0.001	0.79 (0.68, 0.92)	0.002
Adjusted model 4				
Dietary potassium intake quartile				
Q1	Ref		Ref	
Q2	0.80 (0.70, 0.93)	0.003	0.78 (0.60, 1.03)	0.079
Q3	0.80 (0.68, 0.94)	0.006	0.74 (0.53, 1.02)	0.062
Q4	0.66 (0.54, 0.80)	<0.001	0.53 (0.36, 0.79)	0.002
Per SD increment	0.84 (0.78, 0.90)	<0.001	0.76 (0.65, 0.90)	0.001
Adjusted model 5				
Dietary potassium intake quartile				
Q1	Ref		Ref	
Q2	0.81 (0.68, 0.97)	0.019	0.85 (0.64, 1.13)	0.257
Q3	0.82 (0.67, 1.00)	0.051	0.76 (0.54, 1.07)	0.116
Q4	0.69 (0.54, 0.88)	0.003	0.58 (0.38, 0.88)	0.011
Per SD increment	0.88 (0.80, 0.96)	0.005	0.81 (0.69, 0.96)	0.015

NAFLD, nonalcoholic fatty liver disease; AHF, advanced hepatic fibrosis; crude model was not adjusted. Adjusted model 1 adjusted for age, sex, race, education level, and ratio of family income to poverty. Adjusted model 2 adjusted for model 1 + smoking status, work activities status, and recreational activities status. Adjusted model 3 adjusted for model 2 + dietary energy intake, dietary protein intake, dietary alcohol intake, dietary sugar intake, dietary fat intake, dietary cholesterol intake, dietary sodium intake, and dietary zinc intake. Adjusted model 4 adjusted for model 3 + hypertension status, and diabetes status. Adjusted model 5 adjusted for model 4 + uric acid, fasting glucose, glycohemoglobin, total cholesterol, HDL cholesterol, creatinine, and BMI.

**Table 3:** Interactive effect of dietary vitamin C intake and dietary potassium intake on NAFLD.

Models	Dietary vitamin C intake				*P* interaction
	Low		High		
	OR (95% Cl)	*P* value	OR (95% Cl)	*P* value	
Crude model					0.0008
Dietary potassium intake quartile					
Q1	Ref		0.97 (0.79, 1.18)	0.7434	
Q2	1.00 (0.87, 1.15)	0.9749	0.77 (0.66, 0.90)	0.0012	
Q3	1.11 (0.95, 1.29)	0.1972	0.85 (0.74, 0.98)	0.0209	
Q4	1.37 (1.13, 1.66)	0.0011	0.75 (0.66, 0.86)	<0.0001	
Adjusted model I					0.0012
Dietary potassium intake quartile					
Q1	Ref		0.99 (0.81, 1.21)	0.9022	
Q2	0.98 (0.85, 1.14)	0.8115	0.79 (0.67, 0.92)	0.0028	
Q3	1.05 (0.90, 1.24)	0.527	0.84 (0.73, 0.97)	0.0191	
Q4	1.26 (1.03, 1.54)	0.0232	0.71 (0.62, 0.82)	<0.0001	
Adjusted model II					0.0118
Dietary potassium intake quartile					
Q1	Ref		1.07 (0.84, 1.37)	0.5667	
Q2	0.91 (0.76, 1.09)	0.309	0.85 (0.70, 1.03)	0.1033	
Q3	1.01 (0.82, 1.25)	0.8918	0.89 (0.74, 1.08)	0.2449	
Q4	1.14 (0.86, 1.51)	0.3515	0.72 (0.58, 0.89)	0.0024	

NAFLD, nonalcoholic fatty liver disease. Crude model was not adjusted. Adjusted model 1 adjusted for age, sex, race, education level, and ratio of family income to poverty. Adjusted model 2 adjusted for model 1 + smoking status, work activities status, recreational activities status, dietary energy intake, dietary protein intake, dietary alcohol intake, hypertension status, diabetes status, uric acid, fasting glucose, glycohemoglobin, total cholesterol, HDL cholesterol, and creatinine.

**Table 4:** Interactive effect of dietary vitamin C intake and dietary potassium intake on AHF.

Models	Dietary vitamin C intake				*P* interaction
	Low		High		
	OR (95% Cl)	*P* value	OR (95% Cl)	*P* value	
Crude model					0.7800
Dietary potassium intake quartile					
Q1	Ref		0.82 (0.57, 1.19)	0.2954	
Q2	0.86 (0.66, 1.13)	0.2929	0.85 (0.63, 1.14)	0.2797	
Q3	0.76 (0.56, 1.04)	0.0821	0.78 (0.60, 1.03)	0.0822	
Q4	0.70 (0.48, 1.01)	0.0575	0.60 (0.46, 0.79)	0.0003	
Adjusted model I					0.8029
Dietary potassium intake quartile					
Q1	Ref		0.84 (0.55, 1.26)	0.3955	
Q2	1.00 (0.73, 1.36)	0.9938	0.85 (0.61, 1.18)	0.3282	
Q3	0.85 (0.59, 1.21)	0.3585	0.74 (0.54, 1.01)	0.0594	
Q4	0.93 (0.61, 1.41)	0.7224	0.62 (0.45, 0.85)	0.0033	
Adjusted model II					0.8713
Dietary potassium intake quartile					
Q1	Ref		0.82 (0.52, 1.29)	0.3803	
Q2	0.93 (0.65, 1.33)	0.6992	0.78 (0.54, 1.14)	0.203	
Q3	0.78 (0.51, 1.20)	0.265	0.70 (0.49, 1.02)	0.0621	
Q4	0.74 (0.43, 1.28)	0.2803	0.51 (0.33, 0.80)	0.0031	

AHF, advanced hepatic fibrosis. Crude model was not adjusted. Adjusted model 1 adjusted for age, sex, race, education level, and ratio of family income to poverty. Adjusted model 2 adjusted for model 1 + smoking status, work activities status, recreational activities status, dietary energy intake, dietary protein intake, dietary alcohol intake, hypertension status, diabetes status, uric acid, fasting glucose, glycohemoglobin, total cholesterol, HDL cholesterol, and creatinine.

## Data Availability

The dataset supporting the conclusions of this article is available in the NHANES repository https://www.cdc.gov/nchs/nhanes/index.htm.

## References

[1] Huh J. H., Lee K. J., Lim J. S. (2015). High dietary sodium intake assessed by estimated 24-h urinary sodium excretion is associated with NAFLD and hepatic fibrosis. *PLoS One*.

[2] Singh S., Allen A. M., Wang Z., Prokop L. J., Murad M. H., Loomba R. (2015). Fibrosis progression in nonalcoholic fatty liver vs nonalcoholic steatohepatitis: a systematic review and meta-analysis of paired-biopsy studies. *Clinical Gastroenterology and Hepatology: The Official Clinical Practice Journal of the American Gastroenterological Association*.

[3] Pais R., Charlotte F., Fedchuk L. (2013). A systematic review of follow-up biopsies reveals disease progression in patients with non-alcoholic fatty liver. *Journal of Hepatology*.

[4] Pouwels S., Sakran N., Graham Y. (2022). Non-alcoholic fatty liver disease (NAFLD): a review of pathophysiology, clinical management and effects of weight loss. *BMC Endocrine Disorders*.

[5] Duell P. B., Welty F. K., Miller M. (2022). Nonalcoholic fatty liver disease and cardiovascular risk: a scientific statement from the American heart association. *Arteriosclerosis, Thrombosis, and Vascular Biology*.

[6] Weaver C. M. (2013). Potassium and health. *Advances in Nutrition*.

[7] Sun K., Lu J., Jiang Y. (2014). Low serum potassium level is associated with nonalcoholic fatty liver disease and its related metabolic disorders. *Clinical Endocrinology*.

[8] Ma Y., He F. J., Sun Q. (2022). 24-Hour urinary sodium and potassium excretion and cardiovascular risk. *New England Journal of Medicine*.

[9] Tabbaa A., Shaker M., Lopez R., Hoshemand K., Nobili V., Alkhouri N. (2015). Low serum potassium levels associated with disease severity in children with nonalcoholic fatty liver disease. *Pediatric gastroenterology, hepatology and nutrition*.

[10] Zolfaghari H., Askari G., Siassi F., Feizi A., Sotoudeh G. (2016). Intake of nutrients, fiber, and sugar in patients with nonalcoholic fatty liver disease in comparison to healthy individuals. *International Journal of Preventive Medicine*.

[11] Azadbakht L., Mirmiran P., Esmaillzadeh A., Azizi T., Azizi F. (2005). Beneficial effects of a Dietary Approaches to Stop Hypertension eating plan on features of the metabolic syndrome. *Diabetes Care*.

[12] Lykkesfeldt J., Michels A. J., Frei B., Vitamin C. (2014). *Advances in Nutrition*.

[13] Dehghan M., Akhtar-Danesh N., McMillan C. R., Thabane L. (2007). Is plasma vitamin C an appropriate biomarker of vitamin C intake? A systematic review and meta-analysis. *Nutrition Journal*.

[14] Luo X., Zhang W., He Z. (2021). Dietary vitamin C intake is associated with improved liver function and glucose metabolism in Chinese adults. *Frontiers in Nutrition*.

[15] Ivancovsky-Wajcman D., Fliss-Isakov N., Salomone F. (2019). Dietary vitamin E and C intake is inversely associated with the severity of nonalcoholic fatty liver disease. *Digestive and Liver Disease: Official Journal of the Italian Society of Gastroenterology and the Italian Association for the Study of the Liver*.

[16] Zeng Q., Zhao L., Meng C. (2020). Prophylactic and therapeutic effects of different doses of vitamin C on high-fat-diet-induced non-alcoholic fatty liver disease in mice. *Biomedicine and pharmacotherapy = Biomedecine and pharmacotherapie*.

[17] Chen H. K., Luo J., Li X. J., Liao W. Z., Hu Y. Q., Guo X. G. (2023). Serum folate associated with nonalcoholic fatty liver disease and advanced hepatic fibrosis. *Scientific Reports*.

[18] Liu X. H., Chen H. K., Luo J. (2024). Potassium affects the association between dietary intake of vitamin C and NAFLD among adults in the United States. *PLoS One*.

[19] Cueto-Galán R., Barón F. J., Valdivielso P. (2017). Changes in fatty liver index after consuming a Mediterranean diet: 6-year follow-up of the PREDIMED-Malaga trial. *Medicina Clínica*.

[20] (2016). EASL-EASD-EASO Clinical Practice Guidelines for the management of non-alcoholic fatty liver disease. *Diabetologia*.

[21] Bedogni G., Bellentani S., Miglioli L. (2006). The Fatty Liver Index: a simple and accurate predictor of hepatic steatosis in the general population. *BMC Gastroenterology*.

[22] Angulo P., Hui J. M., Marchesini G. (2007). Day CP: the NAFLD fibrosis score: a noninvasive system that identifies liver fibrosis in patients with NAFLD. *Hepatology*.

[23] Choi Y., Lee J. E., Chang Y. (2016). Dietary sodium and potassium intake in relation to non-alcoholic fatty liver disease. *The British Journal of Nutrition*.

[24] Crestani S., Gasparotto Júnior A., Marques M. C., Sullivan J. C., Webb R. C., da Silva-Santos J. E. (2014). Enhanced angiotensin-converting enzyme activity and systemic reactivity to angiotensin II in normotensive rats exposed to a high-sodium diet. *Vascular Pharmacology*.

[25] Vio C. P., Gallardo P., Cespedes C., Salas D., Diaz-Elizondo J., Mendez N. (2020). Dietary potassium downregulates angiotensin-I converting enzyme, renin, and angiotensin converting enzyme 2. *Frontiers in Pharmacology*.

[26] Ahmadian E., Pennefather P. S., Eftekhari A., Heidari R., Eghbal M. A. (2016). Role of renin-angiotensin system in liver diseases: an outline on the potential therapeutic points of intervention. *Expert Review of Gastroenterology and Hepatology*.

[27] Kato J., Koda M., Kishina M. (2012). Therapeutic effects of angiotensin II type 1 receptor blocker, irbesartan, on non-alcoholic steatohepatitis using FLS-ob/ob male mice. *International Journal of Molecular Medicine*.

[28] Bataller R., Gäbele E., Schoonhoven R. (2003). Prolonged infusion of angiotensin II into normal rats induces stellate cell activation and proinflammatory events in liver. *American Journal of Physiology-Gastrointestinal and Liver Physiology*.

[29] Houston M. C. (2011). The importance of potassium in managing hypertension. *Current Hypertension Reports*.

[30] Ekmekcioglu C., Elmadfa I., Meyer A. L., Moeslinger T. (2016). The role of dietary potassium in hypertension and diabetes. *Journal of Physiology and Biochemistry*.

[31] Hong T., Chen Y., Li X., Lu Y. (2021). The role and mechanism of oxidative stress and nuclear receptors in the development of NAFLD. *Oxidative Medicine and Cellular Longevity*.

[32] Arroyave-Ospina J. C., Wu Z., Geng Y., Moshage H. (2021). Role of oxidative stress in the pathogenesis of non-alcoholic fatty liver disease: implications for prevention and therapy. *Antioxidants*.

[33] Han M. S., Jung D. Y., Morel C. (2013). JNK expression by macrophages promotes obesity-induced insulin resistance and inflammation. *Science (New York, NY)*.

[34] Coelho J. M., Cansanção K., Perez R. M. (2020). Association between serum and dietary antioxidant micronutrients and advanced liver fibrosis in non-alcoholic fatty liver disease: an observational study. *PeerJ*.

[35] Frei B., England L., Ames B. N. (1989). Ascorbate is an outstanding antioxidant in human blood plasma. *Proceedings of the National Academy of Sciences of the United States of America*.

[36] Lee S. W., Lee Y. J., Baek S. M. (2022). Mega-dose vitamin C ameliorates nonalcoholic fatty liver disease in a mouse fast-food diet model. *Nutrients*.

[37] Laurent A., Nicco C., Tran Van Nhieu J. (2004). Pivotal role of superoxide anion and beneficial effect of antioxidant molecules in murine steatohepatitis. *Hepatology*.

[38] McRae M. P. (2008). Vitamin C supplementation lowers serum low-density lipoprotein cholesterol and triglycerides: a meta-analysis of 13 randomized controlled trials. *Journal of chiropractic medicine*.

[39] Valdecantos M. P., Pérez-Matute P., Quintero P., Martínez J. A. (2010). Vitamin C, resveratrol and lipoic acid actions on isolated rat liver mitochondria: all antioxidants but different. *Redox Report: Communications in Free Radical Research*.

[40] Xie R., Xiao M., Li L. (2022). Association between SII and hepatic steatosis and liver fibrosis: a population-based study. *Frontiers in Immunology*.

[41] Xie R., Zhang Y. (2023). Associations between dietary flavonoid intake with hepatic steatosis and fibrosis quantified by VCTE: evidence from NHANES and FNDDS. *Nutrition, Metabolism, and Cardiovascular Diseases: Nutrition, Metabolism, and Cardiovascular Diseases*.

[42] Hoy M. K., Goldman J. D. (2010). Potassium intake of the U.S. Population: what we eat in America, NHANES 2009-2010. *FSRG Dietary Data Briefs. Beltsville (MD)*.

